# 

**Published:** 1889-06

**Authors:** 


### CONTENTS FOR JUNE, 1889.

PAGE.

ORIGINAL COMMUNICATIONS.

The Principles of Treatment in Pulmonary Tuberculosis, with Some Observations on Its Etiology. By HERMANN M. BIGGS, M. D., . . . . . . . . . . . . . . 633
The Diagnostic Tampon: Its Value in the Recognition of Chronic Endometritis. By DR. B. S. SCHULTZE, . . . . . . . . . . . . . . . . . . . . . . . . . . 649
"Boo Khar"—India Fever. By ALICE B. CONDICT, M. D., . . . . . . . . . . 665
The Treatment of Locomotor Ataxia by Suspension. By WM. C. KRAUSS, B. S., M. D., . . . . . . . . . . . . . . . . . . . . . . . . . . . . . 669

NEW INSTRUMENTS.

A Mechanical Nasal Saw. By FRANK HAMILTON POTTER, M. D., . . . . . . . . 671

EDITORIAL.

A Reviewer Reviewed, . . . . . . . . . . . . . . . . . . . . . . . . 673
The Jenks Prize Essay, . . . . . . . . . . . . . . . . . . . . . . . 680

OBITUARY.

Edward Tobie, M. D., . . . . . . . . . . . . . . . . . . . . . . . . 693

PERSONAL.

Dr. J. B. Andrews—Dr. R. Stansbury Sutton—Dr. Roswell Park—Dr. J. M. Matthews, . . . . . . . . . . . . . . . . . . . . . . . . . . . . . 694
Dr. A. A. Hubbell—Dr. Wm. C. Krauss—Drs. George S. Peck and A M. Clark—Dr. A. E. Persons, . . . . . . . . . . . . . . . . . . . . . . . . . . 695

SOCIETY MEETINGS.

The Annual Meeting of the American Medical Editors' Association, . . . . . . . 695
The American Public Health Association, . . . . . . . . . . . . . . . . . 705

NEW JOURNALS.

*The St. Louis Polyclinic—The Kansas Medical Journal—Philadelphia Times and Register*, . . . . . . . . . . . . . . . . . . . . . . . . . . . 696

REMOVALS.

Dr. Cyrus A. Allen—Dr. J. Falk—Dr. J. D. Flagg, . . . . . . . . . . . . . 697

BOOK REVIEWS.

Lectures on Nervous Diseases (RANNEY), . . . . . . . . . . . . . . . . . 697
The American Armamentarium Chirurgicum, . . . . . . . . . . . . . . . . . 699
Wood's Medical and Surgical Monographs, . . . . . . . . . . . . . . . . . 701
American Resorts, with Notes upon their Climate, . . . . . . . . . . . . . 702
Atlas of Venereal and Skin Diseases, . . . . . . . . . . . . . . . . . . 703
Diseases and Injuries of the Ear, . . . . . . . . . . . . . . . . . . . 704

LITERARY AND OTHER NOTES.

The Daggett Examining Table, . . . . . . . . . . . . . . . . . . . . . 705
The Annals of Surgery—Wampole's Preparations—The Cosmopolitan, . . . . . 706
The Johns Hopkins Hospital—Now Ready—McCray's Liquid Dovers—Books and Pamphlets Received, . . . . . . . . . . . . . . . . . . . . . . . . . . 707

